# Periodontal Management of Cyclosporin A-Induced Gingival Overgrowth: A Nonsurgical Approach

**DOI:** 10.1155/2019/8609547

**Published:** 2019-04-11

**Authors:** Rayhana Malek, Bouchra El Houari, Jamila Kissa

**Affiliations:** Clinical Department of Periodontology, School of Dentistry, Hassan II University, Casablanca, Morocco

## Abstract

Gingival overgrowth is a major and frequent unwanted effect accompanying the chronic usage of antihypertensive, anticonvulsant, and immunosuppressant drugs. The expression and the severity of this tissue-specific condition are influenced by a variety of factors, mainly drug and periodontal variables. Such increased volume of gingiva may compromise normal oral functions, aesthetics in addition to the patients' ability to practice optimal oral hygiene. The management of gingival overgrowth includes nonsurgical approach, surgical approach, or both of them for severe cases of gingival overgrowth as well as drug withdrawal. This case report illustrates a successful nonsurgical management of a 21-year-old patient with cyclosporin A-induced gingival overgrowth who experienced a total regression of the gingival enlargement without any surgical procedure or drug substitution. And it highlights therefore the key role of supportive periodontal therapy in maintaining good and stable outcomes over 2 years of follow-up.

## 1. Introduction

Gingival enlargement or gingival overgrowth (GO) is the preferred term for all medication-related gingival lesions previously termed gingival hyperplasia or gingival hypertrophy [1]. It is a frequent side effect associated with three major drug groups: anticonvulsants, calcium channel blockers, and immunosuppressants especially cyclosporin A (CsA) [1].

The prevalence of this gingival overgrowth varies between drugs, and its expression is influenced by a variety of risk factors [2]. This prevalence is from 6 to 15% for nifedipine, about 50% for phenytoin, and is from 25% to 30% in adult patients and >70% in children for cyclosporin A [1]. Besides, according to a recent data of Hatahira et al. [3], the reported ratio of CsA-induced gingival overgrowth is 39.4 [3].

Gingival overgrowth normally begins at the interdental papillae and is more frequently found in the anterior segment of the labial surfaces [1].

Although the precise mechanism of this GO remains incomplete, it is probably a result of the interaction between cyclosporin A and its metabolites with susceptible gingival fibroblast cells. Plaque-induced gingival inflammation appears to enhance this interaction [4].

This increased gingival volume is often the cause of difficulties for plaque control and complaints of discomfort, pain, and aesthetic prejudice [1, 3].

Different treatment options can be suggested to manage GO; they can be categorized as nonsurgical approach and surgical approach. The nonsurgical approach is aimed at reducing the inflammatory component in gingival tissue. The surgical approach eliminates the fibrotic component of the gingival tissue when it is severe and persists after the nonsurgical therapy [5].

In this case report, we present a successful nonsurgical management and good middle-term outcome of cyclosporin A-induced gingival overgrowth.

## 2. Case Report

A 21-year-old woman was consulted in February 2015 for bleeding gingival enlargement evolving for 12 months. She complained of esthetics, discomfort, and difficulties of plaque control. According to medical history, the patient had received a kidney transplantation 2 years earlier (2013). She has been administrating a daily immune suppressor treatment based on cyclosporin A 125 mg, prednisolone 5 mg, and mycophenolate mofetil 500 mg per day as a prophylaxis against organ transplant rejection.

The patient had a very poor oral plaque control; the plaque index PI [6] and gingival index GI scores [7] were high which were, respectively, 2 and 2.75.

**Figure 1:**
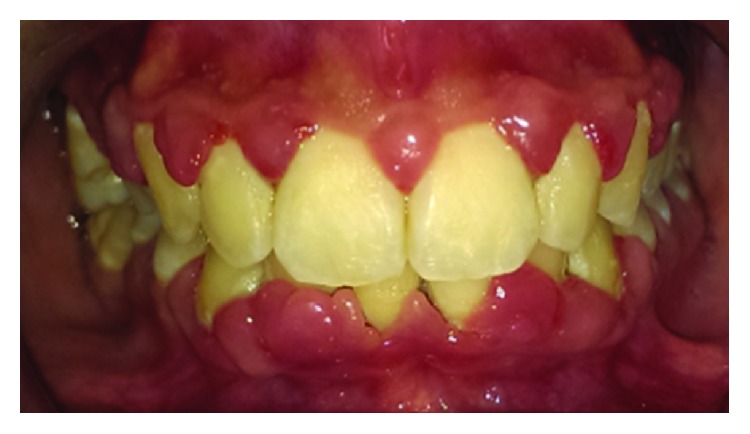
Baseline front view.

**Figure 2:**
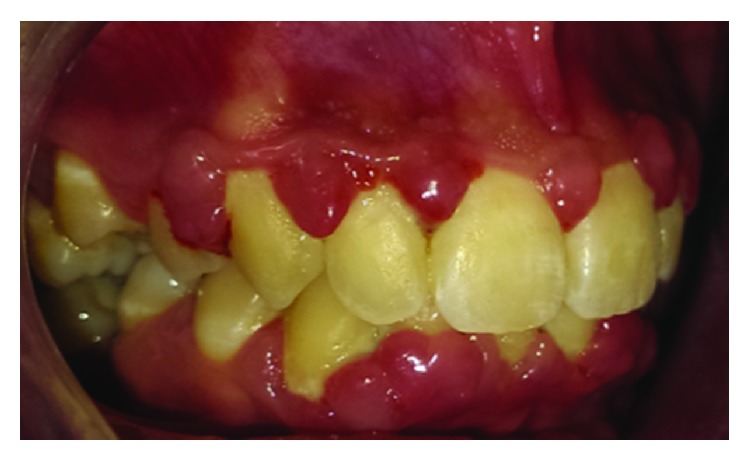
Baseline right side view.

**Figure 3:**
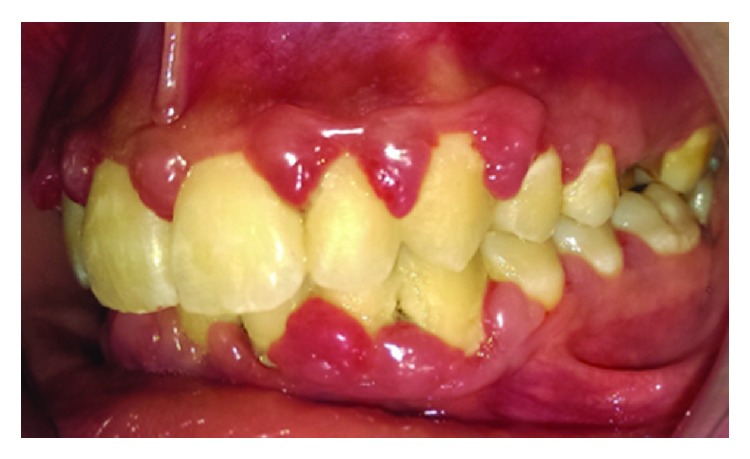
Baseline left side view.

The clinical examination revealed an erythematous, edematous gingival overgrowth localized at the buccal and lingual side of the anterior teeth. The gingival overgrowth appeared as localized nodular enlargement of the interdental papilla (Figures 1–3).

The amount of the gingival overgrowth was obtained according to the GO score of Seymour et al. [8].

A GO score was assigned to each buccal and lingual interdental papilla (gingival unit) of the six anterior upper and lower teeth. Then the sum of the horizontal and the vertical enlargement components was made.

The first component measured the degree of gingival thickening (horizontal enlargement) labially and lingually by means of a three-point scale (0 = normal width, 1 = thickening up to 2 mm, and 2 = thickening of more than 2 mm). The second component measured the extent of encroachment (vertical enlargement) of the gingival tissues on the labial and lingual aspects of adjacent tooth crown; it ranged from 0 to 3 (from no clinical evidence of overgrowth to an overgrowth covering three-fourths of the tooth crown). Likewise, a total of 20 papillae are examined, presenting a potential maximum GO score of 100, which could be expressed as a percentage [8].

The gingival overgrowth is considered as clinically significant if the GO score is ≥30% [9].

In the present case report, the GO score was 30.5%, so that it was classified as clinically significant gingival overgrowth.

A suitable probing revealed deep pockets with negative recessions, due to the gingival overgrowth (indicating coverage of clinical crowns ≥ 2 mm). Underlying calculus was localized mainly at the anterior teeth. The pocket values and clinical attachment loss varied from 5 to 7 mm and from 2 to 3 mm, respectively.

**Figure 4:**
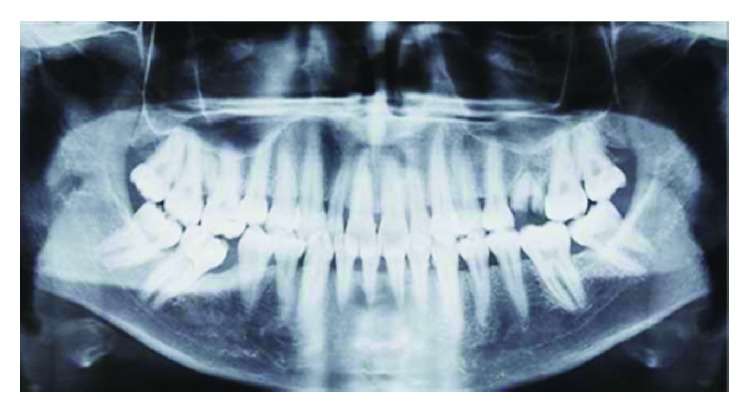
Baseline radiographic examination.

X-ray examination showed a marginal (coronal third) horizontal alveolar bone loss which was more pronounced at the lower incisors (Figure 4). So the patient had a periodontitis beside the gingival enlargement.

The final diagnosis was CsA-induced gingival overgrowth with underlying localized moderate periodontitis stage II grade B. The periodontitis was classified according to the new classification system of periodontal diseases and conditions from the American Academy of Periodontology and the European Federation of Periodontology 2018 [10] (Tables 1 and 2).

The management strategy consisted of a nonsurgical periodontal therapy based, initially, on oral hygiene instruction. On the second-time round, a full-mouth scaling and root planning were performed a week later as well as polishing of all the rough dental surfaces. Extraction of the remaining root of tooth #26 was done at the same appointment.

The treatment was conducted under appropriate antibiotic prophylaxis based on amoxicillin plus clavulanic acid 1 g (intraoral) 2 times per day for 8 days as suggested by the patient's nephrologist. The antibiotic prophylaxis was performed in order to cover the infectious risk related to the systemic health status.

Two months after the periodontal treatment (hygienic phase), the clinical evaluation showed a successful regression of the inflammation and improvement of periodontal parameters. We have noted a reduction of pockets' depth and plaque and gingival index scores which become, respectively, PI: 0.5 and GI: 0.8.

**Figure 5:**
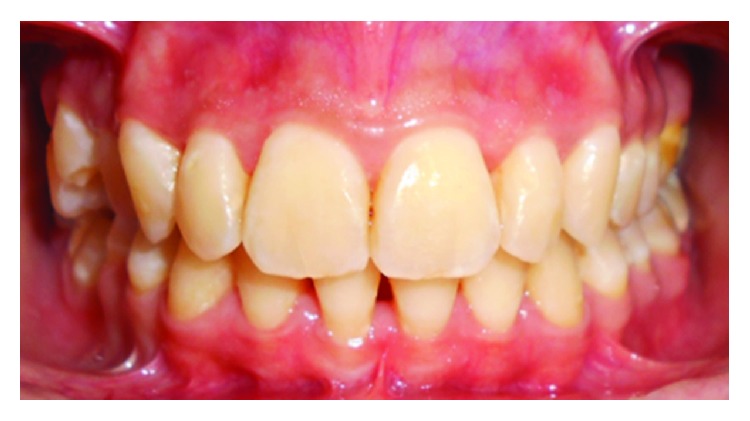
Final front view 2 years post treatment.

**Figure 6:**
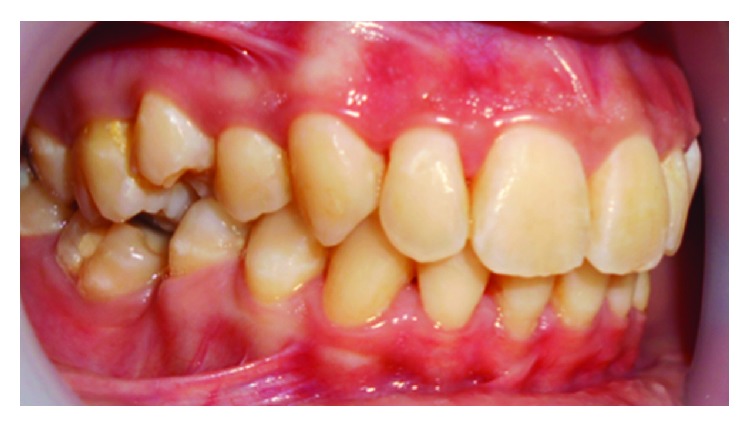
Final right side view 2 years post treatment.

**Figure 7:**
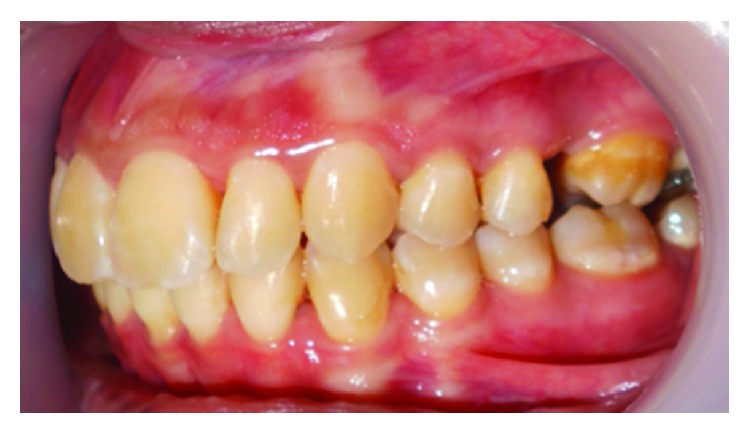
Final left side view 2 years post treatment.

**Figure 8:**
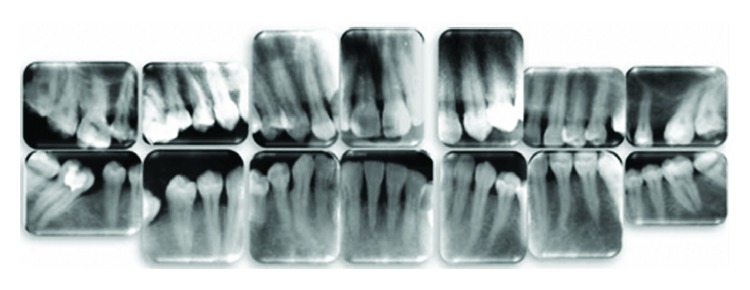
Final periapical radiographs 2 years post treatment.

Thus, a supportive therapy was established including the reinforcement of oral hygiene instruction and full-mouth scaling every 2 months. The whole treatment resulted in the total disappearance of gingival overgrowth without any surgical procedure. The last clinical and X-ray evaluation after 2 years of regular follow-up shows the good stability of the results (Figures 5–8).

## 3. Discussion

Gingival overgrowth (GO) is a well-documented unwanted effect associated with the systemic use of cyclosporin A (CsA). This molecule is an immunosuppressive drug extensively used for the prevention of organ transplant rejection as well as the management of a number of autoimmune conditions [1].

It was reported that six risk factors could modify cyclosporin A-induced gingival overgrowth prevalence and severity. These factors are genetic predisposition, age, gender (young male patients are at greater risk for GO), and drug variables (serum concentration, salivary concentration, and drug dosage) as well as concomitant medication especially calcium channel blockers and periodontal variables such as plaque accumulation and preexisting gingival inflammation [2, 11].

The plaque accumulation is a strong cofactor in the etiology of CsA-induced gingival overgrowth. Indeed, the severity of this gingival enlargement correlates well with poor plaque control [1].

A study of Greenberg et al. [12] showed a statistically significant association between GO and visible plaque accumulation. The median percentage of sites with PI ≥ 2 (visible plaque) was significantly higher among renal transplant patients with GO (42%) than among those without GO (16%; *P* < 0.0001) [12].

The clinical manifestation of gingival enlargement appears frequently within 1 to 3 months after initiation of medications [1]. It may reach a plateau phase at 9 to 12 months [13], as illustrated in the present case report. Besides, a recent data showed that the median time to onset of GO values for immunosuppressants is around 37 days [3].

Gingival overgrowth is more frequently found in the buccal surfaces of the anterior teeth. It is characterized by a growth of the height of gingiva towards the incisal edge of the clinical crown (vertical growth) and then a growth of thickness of the gingiva towards the buccal-lingual (horizontal growth) area which occurs after.

The gingival enlargement begins at the interdental papillae like gingival lobulation; with further progression, the increasing gingiva extends coronally to cover a large amount of the dental crown [1, 13, 14].

This increased susceptibility of the interdental papilla to nodular enlargement in the initial stages of gingival overgrowth may be related to differences in the molecular and cellular composition of different parts of the gingiva. Csiszar et al. [15] reported that the molecular composition of the interdental papilla is distinct from that of the marginal gingiva, suggesting that the cells in the interdental papilla are in an activated state and/or inherently display a specific phenotype resembling wound healing [15].

Although the clinical features of all drug-induced gingival overgrowth seem similar, it was reported that tissues affected by CsA are generally more hyperemic and bleed more readily upon probing [1, 16]. Indeed, a histopathological finding showed that CsA-induced GO is highly inflamed and exhibits little fibrosis than other drug-induced lesions [17].

In the present case report, we could not do any histopathological exploration because of the total regression of the GO after the nonsurgical periodontal therapy. So, we did not have any remaining GO tissue specimen for exploration. We could conclude that the more predominant etiology was probably dental biofilm and calculus.

The exact pathogenic mechanism of CsA-induced gingival overgrowth is still discussed. It seems that this drug and its metabolite disturb the proliferation and the function of the fibroblast cells. Besides, CsA has a synergistic action with proinflammatory and fibrogenic cytokines (Il-1b, Il-6) and interferes with matrix metalloproteinase (MMP) synthesis and function [1, 4].

As not all patients treated with CsA present a gingival overgrowth, it was speculated that this type of GO is related to an individual drug susceptibility. Since gingival fibroblasts may show an individual drug response, fibroblast responders versus fibroblast nonresponders, it is possible that CsA and its metabolite react with a phenotypically distinct subpopulation of gingival fibroblasts [13, 18, 19].

The renal transplant patients are at a higher risk of serious infection because they are under immunosuppressants such as CsA and also corticoids like prednisolone. These drugs suppress the immune system and prevent an organ transplant rejection [20].

So there is usually a recommendation for prophylactic antibiotics, although there is no evidence-based research or guidelines for this. Batiuk et al. [21] and Guggenheimer et al. [22] used and recommended the 1997 American Heart Association endocarditis prevention regimen based on amoxicillin 2 g 1 h preoperatively [21, 22].

Nonsteroidal anti-inflammatory drugs and antibiotics such as erythromycin and clarithromycin are not recommended. They can interfere with cyclosporin and could raise the serum levels, rendering the patient more immunosuppressed than desired [23].

Despite these recommendations, the prophylactic antibiotic protocol should always be made in consultation with the patient's doctor [20], as we did for the management of the present case report.

As far as periodontal treatment is concerned, current treatment options include nonsurgical interventions alone or a combination of nonsurgical and surgical interventions. Nonsurgical approaches include an oral hygiene program, a scaling and root planning, and also the elimination of local irritant factors that enhance plaque accumulation (faulty restorations, broken teeth, or carious lesions).

This periodontal therapy is effective since it can reduce the volume of the gingival enlargement up to 40% [24]. Besides, a study of Aimetti et al. [25] showed that nonsurgical periodontal treatment allows a more significant reduction of the gingival overgrowth. It also avoids the need of surgical therapy even 12 months after nonsurgical treatment and maintenance [25]. It was demonstrated that the clinical control of inflammation and GO by nonsurgical periodontal treatment results histologically both in lowering of inflammatory infiltrate and in changes in connective tissue composition [26].

The use of adjuvant antibiotic therapy has been suggested. Thus, a systematic review of Clementini et al. [27] revealed that a 5-day course of azithromycin with scaling and root planning reduced the degree of the gingival overgrowth compared to metronidazole [27]. However, another study by Mesa et al. [28] confirmed that both molecules could be effective on concomitant bacterial overinfection rather than CsA-induced GO regression [28].

Drug withdrawal or substitution, such as switching from CsA to tacrolimus (FK 506), is another approach. It can reduce the severity of overgrowth and the need for surgical intervention [29]. It was reported that the odds of having gingival enlargement were five times higher among renal transplant patients on cyclosporin than among those on tacrolimus [12].

Although a substitution in medication may improve the gingival tissues, it does not necessarily lead to the complete resolution of the overgrowth [30]. Nonetheless, if such a strategy is considered, the dentist must liaise with the patient's physician to review their current medication.

Recently, the UV phototreatment (UV radiation of –254 nm) has been proposed by Ritchhart and Joy [31]. It might be a viable nonsurgical treatment modality as it is based on the activation of fibroblast cell apoptosis [31].

When the gingival enlargement is severe or persists, despite drug substitution attempts and good plaque control, surgical correction is advocated. It includes scalpel gingivectomy, flap surgery, electrosurgery, or laser excision [5]. Conventional gingivectomy remains the treatment of choice because it leads to a smoother postsurgical gingival surface. Electrosurgery and laser excision produce a good and adequate hemostasis in such inflamed overgrown gingival tissues [5]. But it was shown that the laser excision resulted in a much lower rate of recurrence and provided more comfort for patients compared to flap surgery and scalpel gingivectomy [32].

The high recurrence rate of gingival overgrowth remains a problem arising from the chronic usage of CsA and other drugs [18]. According to the data of Ilgenli et al. [33], the recurrence rate accounted for 34% of cases and could occur within 18 months even after surgical therapy regardless of the drug. Besides, poor plaque control, gingival inflammation, and poor patient compliance with maintenance visits were found to be significant determinants of this relapse [33].

So, the regular supportive periodontal therapy is effective in resolving the inflammation and the gingival overgrowth and in eliminating the need for surgical treatment [25]. That was highlighted in this case report with more than 24 months of regular follow-up.

## 4. Conclusion

Gingival overgrowth is a serious side effect accompanying the use of cyclosporin A. The diagnosis is easy according to medical history and intraoral examination of the patient. Bacterial plaque accumulation is the major risk factor that may initiate and exacerbate the increasing volume of gingival tissues. The treatment options can be categorized as nonsurgical therapy alone or a combination of nonsurgical and surgical therapy. All these approaches have been attempted to either reduce or eliminate gingival enlargement and its pockets. Finally, good compliance with oral hygiene practices and maintenance visits remains crucial because it allows better and stable outcomes after the treatment and prevents from gingival overgrowth recurrence.

## Figures and Tables

**Table 1:** Periodontitis stage according to the 2018 new classification.

Periodontitis stage		Stage I	Stage II	Stage III	Stage IV
Severity	Interdental CAL at the site of the greatest loss	1 to 2 mm	3 to 4 mm	≥5 mm	≥5 mm
	Radiographic bone loss	Coronal third (<15%)	Coronal third (15 at 33%)	Extending to the middle or apical third of the root	Extending to the middle or apical third of the root
	Tooth loss	No tooth loss due to periodontitis		Tooth loss due to periodontitis of ≤4 teeth	Tooth loss due to periodontitis of ≥5 teeth

Complexity	Local	Maximum probing depth ≤ 4 mm Mostly horizontal bone loss	Maximum probing depth ≤ 5 mm Mostly horizontal bone loss	(i) In addition to stage II complexity Probing depth ≥ 6 mm (ii) Vertical bone loss ≥ 3 mm (iii) Furcation involvement Class II or III (iv) Moderate ridge defect	In addition to stage III complexity: need for complex rehabilitation due to (i) Masticatory dysfunction (ii) Secondary occlusal trauma (tooth mobility degree ≥ 2) (iii) Severe ridge defect (iv) Bite collapse, drifting, and flaring (v) Less than 20 remaining teeth (10 opposing pairs)

Extent and distribution	Add to stage as descriptor	For each stage, describe extent as localized (<30 % teeth involved), generalized, or molar/incisor pattern.			

**Table 2:** Periodontitis grade according to the 2018 new classification.

Periodontitis grade			Grade A: slow rate of progression	Grade B: moderate rate of progression	Grade C: rapid rate of progression
Primary criteria	Direct evidence of progression	Longitudinal data (radiographic bone loss or CAL)	Evidence of no loss over 5 years	<2 mm over 5 years	>2 mm over 5 years
	Indirect evidence of progression	% bone loss/age	<0.25	0.25 to 1.0	>1.0
		Case phenotype	Heavy biofilm deposits with low levels of destruction	Destruction commensurate with biofilm deposits	Destruction exceeds expectation given biofilm deposits; specific clinical patterns suggestive of periods of rapid progression and/or early onset of disease, e.g., molar/ incisor pattern; lack of expected response of standard bacterial control therapies

Grade modifiers	Risk factors	Smoking	Nonsmoker	Smoker < 10 cigarettes/day	Smoker > 10 cigarettes/day
		Diabetes	Normoglycemic/no diagnosis of diabetes	HbA1c < 7.0% in patients with diabetes	HbA1c > 7.0% in patients with diabetes

Risk of systemic impact of periodontitis	Inflammatory burden	High-sensitivity CRP (hs CRP)	<1 mg/L	1-3 mg/L	>3 mg/L

Biomarkers	Indicators of CAL/bone loss	Saliva, gingival crevicular fluid, and serum	?	?	?
